# A Novel Statistical Approach for Brain MR Images Segmentation Based on Relaxation Times

**DOI:** 10.1155/2015/154614

**Published:** 2015-12-21

**Authors:** Fabio Baselice, Giampaolo Ferraioli, Vito Pascazio

**Affiliations:** ^1^Dipartimento di Ingegneria, Università di Napoli Parthenope, Centro Direzionale di Napoli, Isola C4, 80143 Napoli, Italy; ^2^Dipartimento di Scienze e Tecnologie, Università di Napoli Parthenope, Centro Direzionale di Napoli, Isola C4, 80143 Napoli, Italy

## Abstract

Brain tissue segmentation in Magnetic Resonance Imaging is useful for a wide range of applications. Classical approaches exploit the gray levels image and implement criteria for differentiating regions. Within this paper a novel approach for brain tissue joint segmentation and classification is presented. Starting from the estimation of proton density and relaxation times, we propose a novel method for identifying the optimal decision regions. The approach exploits the statistical distribution of the involved signals in the complex domain. The technique, compared to classical threshold based ones, is able to globally improve the classification rate. The effectiveness of the approach is evaluated on both simulated and real datasets.

## 1. Introduction

In Magnetic Resonance Imaging (MRI) field, tissues segmentation can be helpful in several applications, such as image-guided interventions, surgical planning, and radiotherapy, but also in 2D/3D visualization, studying brain diseases, or clinical drug trials [1–3]. The aim of segmentation consists in identifying the different regions across the imaged slice. A step ahead is the classification which assigns each region to a class; that is, it identifies the involved tissues.

In this paper we restrict the analysis to MR images of brain. In this case, segmentation is a fundamental tool in quantification of white matter lesions in case of drug treatment assessment or in the study of temporal evolution of many disorders, such as multiple sclerosis, schizophrenia, epilepsy, or Alzheimer's disease. In particular, segmentation is able to provide the volumetric analysis of gray matter, white matter, and cerebrospinal fluid and to allow the morphological differences characterization between subjects.

Few decades ago, the manual delineation of MR images by a human expert was the main tool for segmenting tissues. Unfortunately, this approach is characterized by several disadvantages: the accurate delineation of complex 3D anatomical structures was very complex, results had a considerable inter- and intrarater variability, and it was very time consuming [4]. So in last decades big efforts for achieving effective automated procedures have been done [5].

Automatic segmentation techniques belong to two main categories: structural and statistical [6]. The former one is based on the recognition of anatomical shapes across the image, while the latter takes into account the statistical distribution of the acquired data. Among these two categories, the most used approaches are classification-based segmentation, region-based segmentation, and contour-based segmentation. Within this paper, we focus on classification-based approaches, that is, jointly segment and classify tissues across the imaged slice. In this kind of approach, voxels are classified and labeled as belonging to a particular tissue class according to a certain criterion. The simplest method is based on the application of a threshold. While this is a trivial operation, the determination of the proper thresholding value has to be carefully done. Thresholds are applied to a metric, which generally is the Euclidean distance of pixel gray level values. Basic approaches consider the Gaussian mixture model of tissues signal intensities, that is, a one-dimensional problem. If a proper postprocessing is not implemented, such approach produces poor results in case of low Signal to Noise Ratio (SNR) and tissues with similar signal intensities. Moreover, several artifacts could affect the images, such as the intensity inhomogeneity that makes the ranges of the intensities in the regions to segment overlapped [7].

Within this paper we propose a brain joint segmentation and classification algorithm based on proton density (*ρ*) and relaxation times (*T*_1_ and *T*_2_), instead of the acquired gray level image. The idea of exploiting relaxation times for improving segmentation performances is not new, as methods based on single or multiple weighted images have been presented [4, 8]. The main limit of these approaches consists in computing the segmentation in a monodimensional space and eventually joining the results as a postprocessing step. What we propose is a segmentation in a 3D space, jointly based on *ρ*, *T*_1_, and *T*_2_ maps and not on weighted images. The physical parameters are first estimated from multiple acquired images and then used for the segmentation. As each voxel is segmented by considering three values (*ρ*, *T*_1_, and *T*_2_) instead of one (gray level), the approach works projecting each voxel in a 3D space (with coordinates *ρ*, *T*_1_, and *T*_2_) instead of a 1D one (with the gray level value as coordinate), proposing a new distance criterion, often referred to as metric. From a geometrical point of view, the projection of image points in a 3D space instead of a 1D line enlarges the distances between classes, making the segmentation and classification operations more accurate. In particular, the greater distances due to the 3D space are expected to reduce the wrong segmented points percentage. For the proposed approach, the ideal thresholds of the segmentation regions, which in this case are 3D curves, are automatically determined starting from the joint statistical distribution of the *ρ*, *T*_1_, and *T*_2_ estimators. The proposed metric is expected to have potentialities also in different frameworks, such as Magnetic Resonance Fingerprinting, which is capable of estimating proton density, *T*_1_ and *T*_2_, in a single scan [9].

Results on a simulated dataset are used to assess and quantitatively evaluate the proposed methodology. Results on a real dataset are used to show the effectiveness of the approach if compared with a standard distance based threshold technique, its robustness to intensity inhomogeneity fields, and its potentialities.

## 2. Methodology

Let us consider an MRI acquisition system using a Spin Echo imaging sequence. The amplitude of the recorded complex signal after image formation process, that is, after the computation of the 2D Fourier Transform, is related to the tissues parameters *ρ*, *T*_1_, and *T*_2_. By considering a single pixel, that is, one voxel of the slice, its intensity can be written as [10, 11]

(1) $$\begin{array}{c} f\left(\;\theta \;\right)=\rho \exp\left(\;-\frac{T_{E}}{T_{2}}\;\right)\left(\;1-\exp\left(\;-\frac{T_{R}}{T_{1}}\;\right)\;\right), \end{array}$$

where *T*_*E*_ and *T*_*R*_ are the echo and repetition time, respectively, which are two imaging parameters that can be set in the MRI scanner and $\theta ={\left[\begin{array}{ccc} \rho & T_{1} & T_{2} \end{array}\right]}^{T}$ is the vector containing the tissue parameters we are interested in. The acquisition model reported in (1) is related to the noise-free case. Considering noise, in the complex domain the model becomes

(2) $$\begin{array}{c} y=y_{R}+iy_{I}=f\left(\;\theta \;\right)\exp\left(\;i\phi \;\right)+\left(\;n_{R}+in_{I}\;\right), \end{array}$$

where *n*_*R*_ and *n*_*I*_ are the real and imaginary parts of the noise samples, which are distributed as independent circularly Gaussian variables [12], and *ϕ* represents the angle of the complex data [13, 14].

We can estimate ***θ*** by implementing an LS estimator [15, 16]:

(3) $$\begin{array}{c} \hat{\theta }=\arg\;\underset{\theta }{\min}\overset{M}{\underset{k=1}{\sum }}\left(\;y_{k}-f\left(\;\theta \;\right)e^{i\phi }\;\right), \end{array}$$

where *M* is the number of images acquired with different *T*_*E*_/*T*_*R*_ combinations.

As it is largely known from statistical estimation theory, in case of Gaussian noise, the LS estimator corresponds to the Maximum Likelihood (ML) one. So, if *M* is sufficiently large, the estimator becomes unbiased and optimal. This allows us to infer the statistical distribution of the estimated values $\hat{\rho }$, $\hat{T_{1}}$, and $\hat{T_{2}}$. In particular, the estimators will be Gaussian distributed with known means and variances. As the estimators are unbiased the mean values *μ*_*ρ*_, *μ*_*T*_1__, and *μ*_*T*_2__ are equal to the unknown parameters, while, since they are optimal, the variances *σ*_*ρ*_^2^, *σ*_*T*_1__^2^, and *σ*_*T*_2__^2^ coincide with Cramer Rao Lower Bounds (CRLBs). Such bounds are related to the acquisition model and to involved noise and represent the lower achievable variance of any unbiased estimator, that is, a quality metric. In the considered acquisition model, CRLBs can be easily calculated numerically or analytically [17].

Thus, the statistical distributions of the random variables $\hat{\rho }$, $\hat{T_{1}}$, and $\hat{T_{2}}$ are

(4) fρ^ρ^=12πσρ2exp⁡−ρ^−μρ22σρ2,fT1^t1^=12πσT12exp⁡−t1^−μT122σT12,fT2^t2^=12πσT22exp⁡−t2^−μT222σT22.

In case different tissues are imaged within the same slice, the pdfs of each tissue have to be taken into account.

Starting from these distributions, the idea of the presented method consists in exploiting such pdfs, in order to find the optimal decision regions in a 3D space for joint segmentation and classification.

Within this framework, three different decision criteria have been developed, which are presented in the following.

### 2.1. Weighted Distance Based Criterion (WDC)

By considering $\hat{\rho }$, $\hat{T_{1}}$, and $\hat{T_{2}}$ estimators to be independent, we can derive the joint pdf by factorizing the marginal pdfs reported in (4). A detector aimed at the maximization of the likelihood function has been implemented. This is equivalent to the maximization of the joint pdf or to the minimization of the negative exponential part:

(5) arg minn⁡ρ^−μρn22σρ2n+t1^−μT1n22σT12n+t2^−μT2n22σT22n,

where ***μ***_*ρ*_(*n*), ***μ***_*T*_1__(*n*), and ***μ***_*T*_2__(*n*) are the mean values of *ρ*, *T*_1_, and *T*_2_ estimators in case of the *n*th (*n* = 1,…, *N*) class, while **σ**_*ρ*_(*n*), **σ**_*T*_1__(*n*), and **σ**_*T*_2__(*n*) are their variances. In case of brain segmentation, *N* = 3 classes are commonly assumed: white matter (WM), gray matter (GM), and cerebrospinal fluid (CSF).

It can be noted that such criterion consists in assigning to each pixel the class related to the closest segmentation region centroid. This centroid is defined according to reference proton density and relaxation times values of involved tissues, which are reported in literature [18]. The distance is computed by considering as weights for *ρ*, *T*_1_, and *T*_2_ differences the inverse of variances of their estimators (i.e., *σ*_*ρ*_^2^, *σ*_*T*_1__^2^, and *σ*_*T*_2__^2^), which ensures that reliable values have a higher weight. A crucial point is the computation of weights: a good choice could be the Cramer Rao Lower Bounds (CRLBs) [17].

Practically, WDC approach, by evaluating the minimum distance class via (5), is equivalent to finding the class assignment with the highest probability. However, in some cases CRLBs are not the ideal choice because of external sources of noise. Often acquired images suffer from intensity inhomogeneity, which could be related to various factors, such as spatial variations in illumination and imperfections of imaging devices [7, 19, 20]. In the estimation of proton density and relaxation times, only the first one is affected by such problem, since *T*_1_ and *T*_2_ are related to a specific decay and thus are independent of the presence of an intensity bias. In this case, it is more effective to rely more on relaxation times than on the proton density. This can be achieved by applying a coefficient to *σ*_*ρ*_^2^. In other words, the segmentation is conducted by considering the *ρ* distance not as much reliable as *T*_1_ and *T*_2_. The weighting coefficient should be manually applied only in case of evident bias.

### 2.2. Statistical Correlation Based Criterion (StCC)

**Figure 1:**
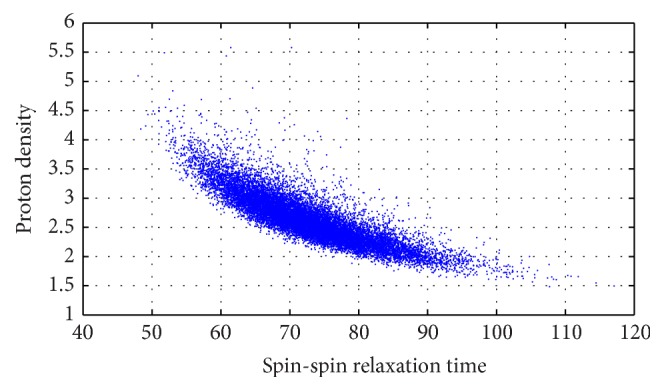
Estimated values projected on the (*ρ*, *T*_2_) plane. *T*_2_ values are in [ms]. Correlation between $\hat{\rho }$ and $\hat{T_{2}}$ estimators is −0.8443.

The WDC is based on the assumption of statistical independence among the three estimators. Such hypothesis can be used in a simplified model. In order to generalize the approach, the mutual correlation among $\hat{\rho }$, $\hat{T_{1}}$, and $\hat{T_{2}}$ has to be taken into account. To give an idea of such correlation, a Monte Carlo simulation has been considered. In each cycle, proton density and spin-spin relaxation time of a voxel are estimated. In Figure 1, one blue point for each Monte Carlo cycle is reported in a 2D Cartesian space. In particular, estimated *T*_2_ is the horizontal axis coordinate, while estimated *ρ* is the vertical one. By looking at these projections of the estimators in (*ρ*, *T*_2_) plane, we can easily note that a nonminimal correlation is present, as the cloud of points is not circular. The exploitation of such correlation leads to statistical correlation based criterion (StCC). In this case the covariance matrix Σ of multivariate Gaussian statistical distribution will be fully populated, leading to the following decision criterion:

(6) $$\begin{array}{c} \underset{n}{\arg\;\min}{\left[\;\hat{x}-\mu \left(\;n\;\right)\;\right]}^{T}\Sigma \left[\;\hat{x}-\mu \left(\;n\;\right)\;\right] \end{array}$$

with

(7) x^=ρ^T1^T2^,μn=μρnμT1nμT2n,Σ=σρ2Covρ,T1Covρ,T2Covρ,T1σT12CovT1,T2Covρ,T2CovT1,T2σT22,

where Cov_*i*,*j*_ is the covariance between the estimators of *i* and *j* parameters. Note that also Σ depends on tissue index *n*, but it has been neglected in the notation.

**Figure 2:**
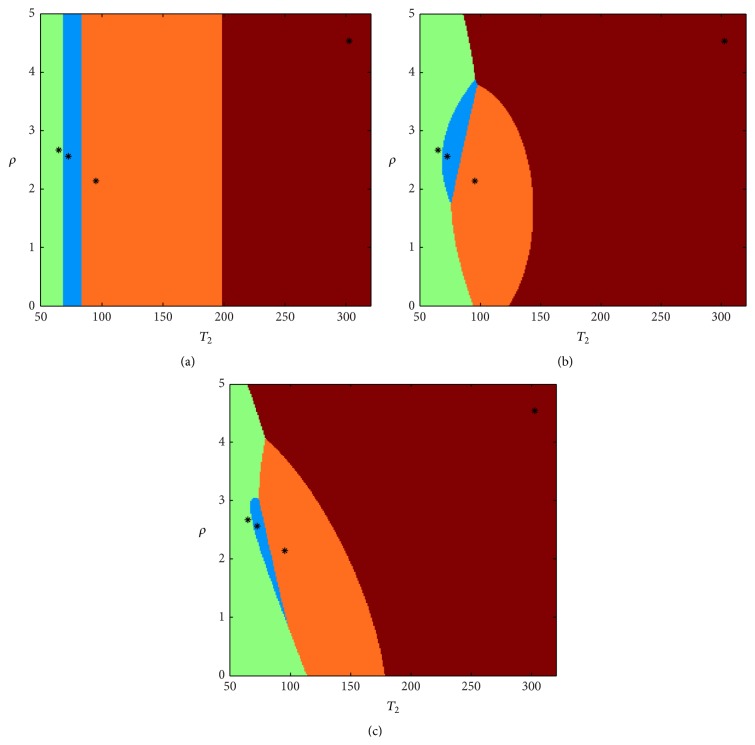
Decision regions computed with the minimum Euclidean distance criterion (a), with WDC (b), and with StCC (c). For each region, the centroid has been marked by an asterisk.

In this case, segmentation and classification are performed by minimizing (6). In order to give an idea of how the classification regions change when adopting such criteria, a comparison is reported in Figure 2. In this figure, four reference tissues are considered with different *ρ*-*T*_2_ combinations (we considered the 2D case instead of the 3D, neglecting *T*_1_ for simplicity); each one is characterized by an asterisk. For each point of the space, that is, for each *ρ*-*T*_2_ pair, the distances from the four reference tissues are computed with the proposed metrics, and it is marked with a color corresponding to the closest class, providing the regions reported in Figure 2.

### 2.3. Spatial Correlation Based Criterion (SpCC)

In order to improve results, a probabilistic regularization criterion has also been considered. Until now, detection and segmentation have been done by considering each pixel independently of all the others, that is, working pixelwise. Here we introduce a spatial dependency between each pixel and its neighborhood with the aim of improving the accuracy.

The spatial correlation based criterion (SpCC) is intended as a refinement of StCC solution. The processing chain consists in a two-step procedure: compute the StCC distances and the related classification and regularize using spatial correlation.

Let us focus on a single pixel and define a neighboring system *Ω*. It collects all the pixels that are close to the considered one. A typical *Ω* is the 8 neighbors, collecting the 8 adjacent pixels (i.e., the considered pixel is positioned in the center of a 3 × 3 window) [21].

Once StCC has been applied, we define *d*_0_ as the minimum distance between the considered pixel and the centroid of the class assigned to it. We, also, define **p**(*n*) as the percentage of the pixels in *Ω* that have been associated with *n*th class. The idea is that if the majority of neighboring pixels belong to the same class, the distance from that class should be shortened in order to regularize the solution. As the distance cannot be negative, the reduction should be at most equal to the minimal distance *d*_0_. Thus, the joint segmentation and classification are carried out by computing:

(8) $$\begin{array}{c} \underset{n}{\arg\;\min}\left\{\;{\left[\;\hat{x}-\mu \left(\;n\;\right)\;\right]}^{T}\Sigma \left[\;\hat{x}-\mu \left(\;n\;\right)\;\right]-p\left(\;n\;\right)d_{0}\;\right\}, \end{array}$$

where the vector **p**(*n*)*d*_0_ is the metric reduction function for all classes. Note that its values are between 0 and *d*_0_, as the probability values in **p**(*n*) are in the [0,1] range.

## 3. Results and Discussion

In order to quantitatively evaluate the advantage of the proposed method with respect to classical unsupervised criteria, a simulated case study has been considered. In particular, a brain slice phantom has been simulated [22]. Four tissues compose the phantom (white matter, subcortical white matter, gray matter, and cerebro spinal fluid) with *ρ* = [2.56,2.67,2.14,4.54], *T*_1_ = [1389,1593,1794,7446] ms, and *T*_2_ = [72.4,65.5,95.2,302] ms. Such values have been measured experimentally with a 3T scanner [23, 24]. A study about the optimal acquisition parameter for the relaxation times estimation can be found in [17].

**Figure 3:**
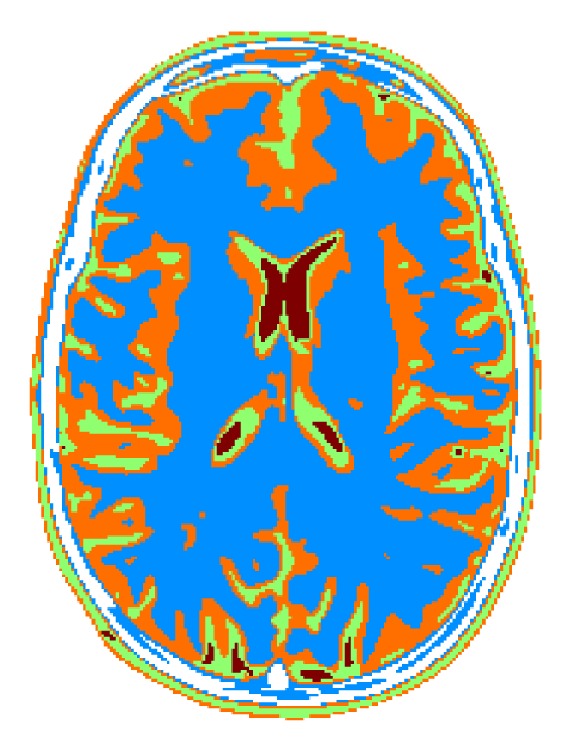
Reference head phantom composed of color coded 4 tissues.

**Figure 4:**
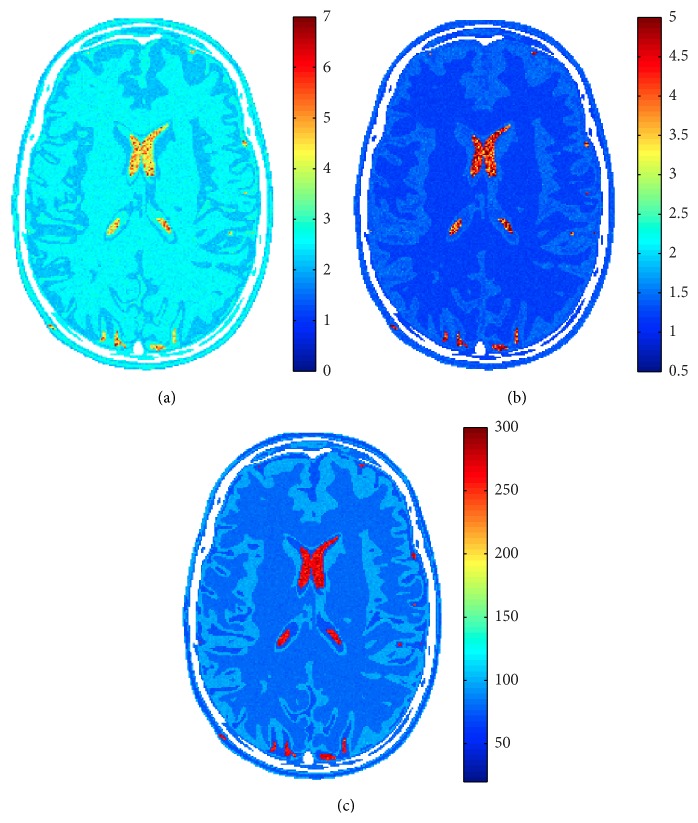
Estimated *ρ* (a), *T*_1_ [s] (b), and *T*_2_ [ms] (c) in case of 4 images and SNR = 30 dB.

The reference phantom is reported in Figure 3, where the four tissues have been coded with blue, orange, green, and red color, respectively. Four images have been generated by simulating Spin Echo imaging sequence, with echo and repetition times of (80,3600), (80,500), (155,3600), and (155,500) [ms], respectively. Gaussian complex noise has been added to the data in order to achieve a mean SNR of 30 dB. Relaxation times have been estimated by using the LS approach [15, 23] of (3), producing *ρ*, *T*_1_, and *T*_2_ estimated maps reported in Figure 4.

**Figure 5:**
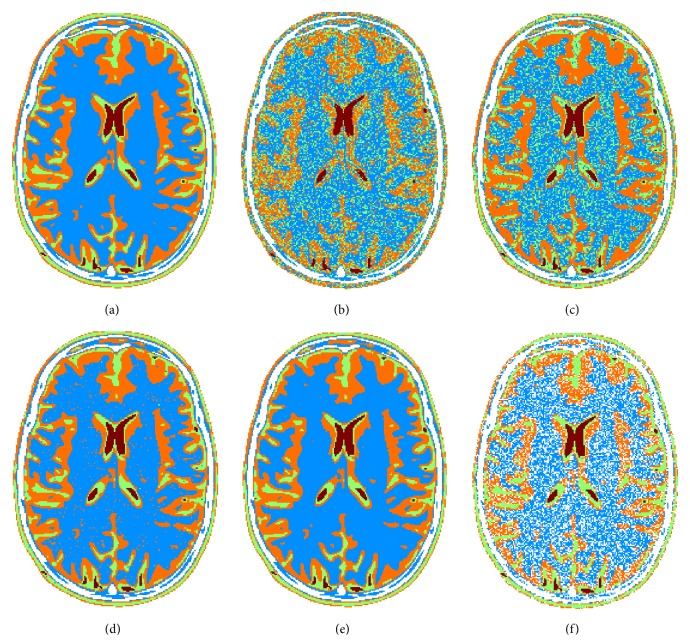
Segmentation and classification results: reference image (a), minimum Euclidean distance approach (b), WDM (c), StCC (d), SpCC (e), and *K*-means approach (f).

The three approaches (WDC, StCC, and SpCC) previously presented have been applied to the considered data. In order to assess the obtained results, classification based on a classical minimum Euclidean distance from the expected values has been reported. Moreover, other methodologies present in literature, working on gray level images, have been investigated: seeded region growing algorithms family has not been considered, as it is supervised [25]; multithreshold maximum entropy has also not been considered due to its difficulty in classifying more than few classes [26]; *K*-means algorithm has been chosen as an interesting reference for the proposed technique [27]. Segmentation results are reported in Figure 5. It has to be considered that reported methodology requires multiple images, as the estimation of proton density and relaxation times is needed, while classical segmentation algorithms work on a single image. In order to have a fair comparison, *K*-means algorithm has been applied to a single image obtained from the estimated $\hat{\rho }$, $\hat{T_{1}}$, and $\hat{T_{2}}$. Such image is characterized by an SNR higher than images of the starting dataset.

**Figure 6:**
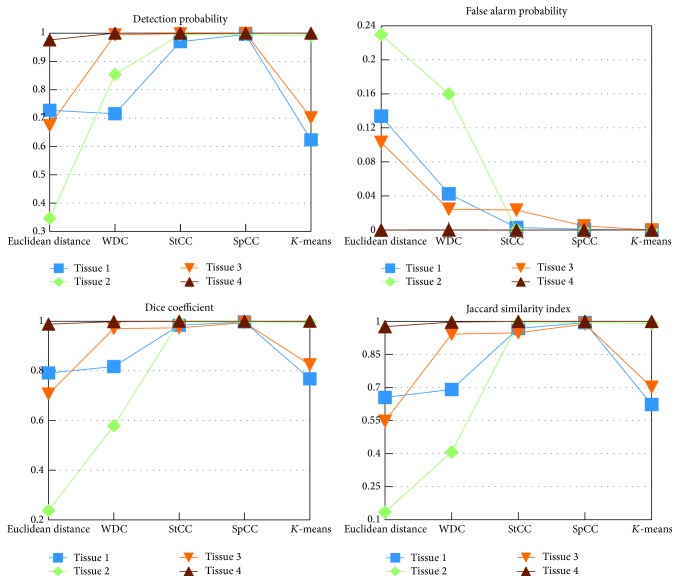
Performance indicator of the four considered methods in case of 4 tissues constituting brain phantom.

**Figure 7:**
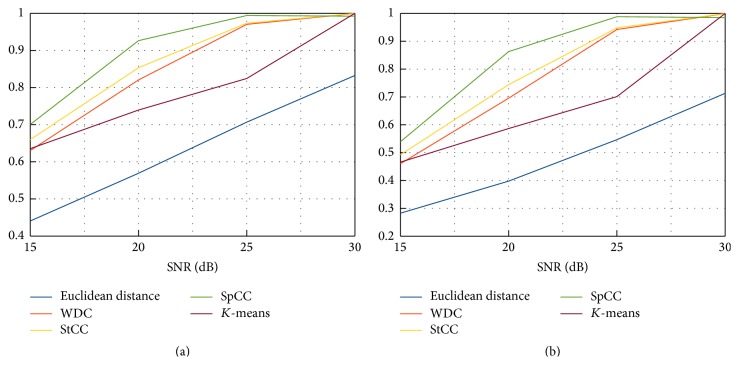
Dice coefficients (a) and Jaccard similarity indexes (b) in case of different SNR for all the considered methods.

In order to give a quantitative performances evaluation, Jaccard indexes [28] and Sørensen-Dice coefficients [29, 30] have been computed and reported in Figure 6. Moreover, the stability of the approaches varying the SNR has been evaluated. Results are reported in Figure 7.

**Figure 8:**
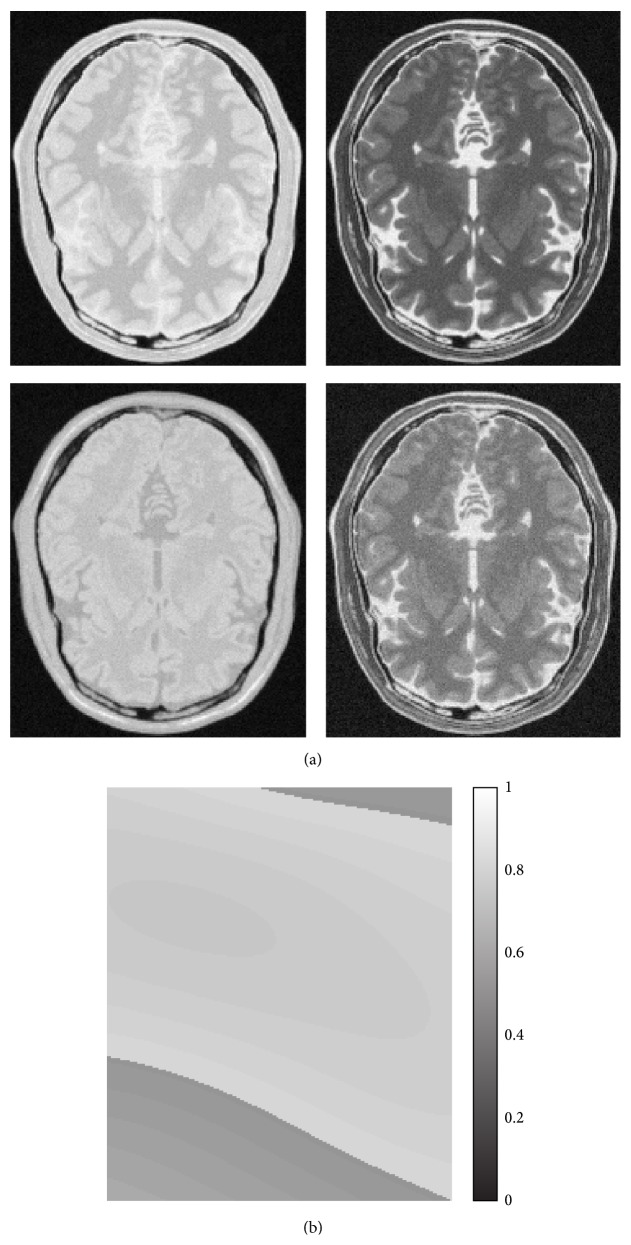
The four Spin Echo images from the BrainWeb database (a). The dataset has a field inhomogeneity of 20% (b).

**Figure 9:**
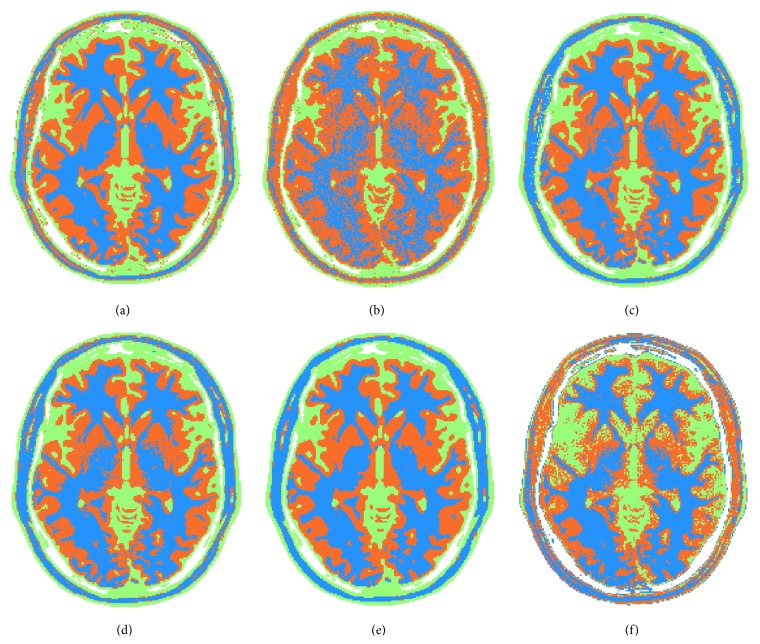
Segmentation and classification results for the BrainWeb phantom: reference image (a), minimum Euclidean distance approach (b), WDM (c), StCC (d), SpCC (e), and *K*-means approach (f).

A second simulated case study has been considered. Four SE images have been downloaded from the BrainWeb (http://www.bic.mni.mcgill.ca/brainweb/) public archive with echo and repetition times of (20,4000), (100,4000), (20,2000), and (100,2000) [ms] [31]. Reference *ρ*, *T*_1_, and *T*_2_ images have been obtained by performing the estimation in a noise-free test case. As a second step, an SNR equal to 30 dB together with a 20% intensity inhomogeneity field has been considered. The four images of the dataset are reported in Figure 8, together with the intensity inhomogeneity field. The considered segmentation approaches have been applied, producing results in Figure 9. In this case, a weight factor has been applied to the CRLB of *ρ* in order to improve the robustness taking into account the inhomogeneity fields (see Section 2.1).

**Figure 10:**
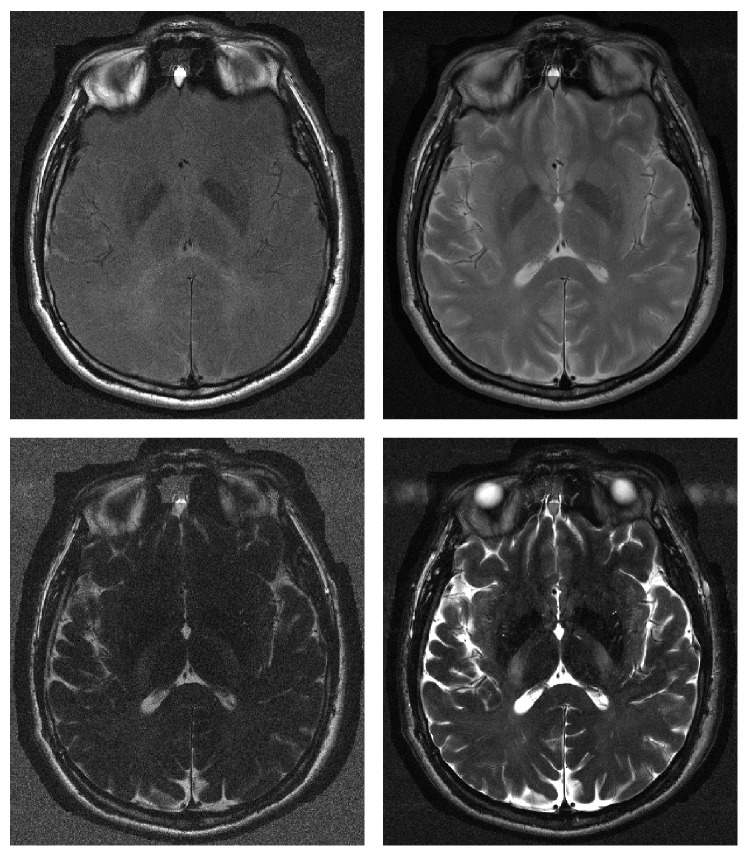
The four Spin Echo images composing the real dataset.

**Figure 11:**
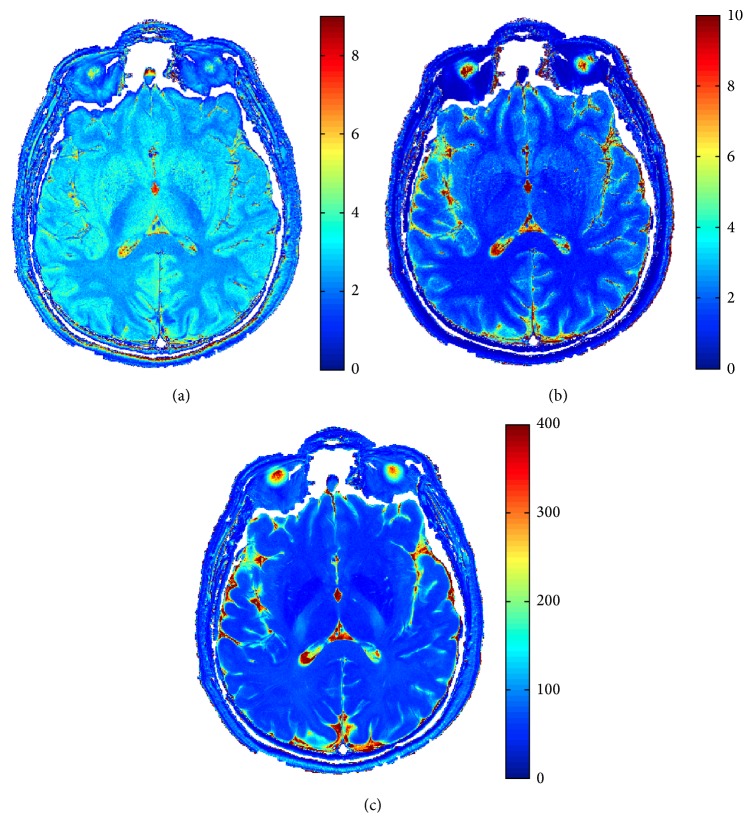
Real dataset estimated *ρ* (a), *T*_1_ [s] (b), and *T*_2_ [ms] (c) in case of 4 images.

Considered methodologies have also been tested on a real case. A male 31-year-old healthy volunteer has been considered. 4 Spin Echo images of a brain slice have been acquired with a 3T Philips Achieva MRI scanner. Acquisition details are reported in Table 1. In Figure 10 the acquired images are reported, while in Figure 11 the estimated proton density and relaxation times maps are shown. In this case, three segmentation classes have been considered, namely, white matter (WM), gray matter (GM), and cerebrospinal fluid (CSF).

Simulated case results of Figure 5 clearly show that the more the statistical distribution of the data is exploited, the better the segmentation and classification accuracy is achieved. Specifically, the improvement can be evaluated by comparing the minimum Euclidean distance based classification (Figure 5(b)) with the WDC (Figure 5(c)) and with StCC (Figure 5(d)). In the latter approach, edges are well retrieved, small structures are preserved, and globally all the regions are correctly classified. Such trends are confirmed in all the synthetic quality criteria that have been chosen and reported in Figure 6, that is, detection probability, false alarm probability, Dice coefficient, and Jaccard similarity index. Errors appear mainly in the blue class, where some wrong isolated orange spots are present. This can be explained considering the closeness of blue and orange classes from Figure 2(c). In order to improve the capability of correctly discriminating blue and orange tissues, the spatial regularization criteria (SpCC) are applied, producing results of Figure 5(e). It is evident that most of isolated spots have been correctly classified without decreasing the segmentation performances of the other classes.

In order to give a reference, *K*-means methodology segmentation results are reported in Figure 5(f). With respect to proposed approach, some points are missing mainly in the blue and orange tissues, which are the most difficult to be discriminated, while good detection performances are achieved with red and green ones. Graphs of Figure 6 confirm such behavior. In particular, *K*-means is characterized by lower detection probability values compared to SpCC for blue and orange classes. On the other hand, SpCC shows good performances for all the four considered classes.

Results about robustness with respect to SNR are reported in Figure 7. In particular, Dice coefficients and Jaccard similarity indexes have been computed in case of Tissue 3 for the considered approaches with an SNR varying from 15 dB to 30 dB. The two graphs are similar, both confirming, as expected, a positive trend for all methodologies. Globally, SpCC is capable of good performances for the SNR values within the considered range, with the best improvement over the others in case of 20 dB. Moreover, at 25 dB a saturation appears, with results similar to 30 dB. On the other hand, the *K*-means approach reaches good performances only in case of high SNR values, being very sensitive to noise at 25 dB and below.

Let us consider results related to the BrainWeb dataset reported in Figure 9. An intensity inhomogeneity affects such dataset, mainly in the bottom left region. It can be seen that *K*-means algorithm segmentation is deeply affected by the intensity inhomogeneity field, in particular where it is more severe, Figure 9(e), as in that region the green tissue is almost never detected. On the other hand, the performances of proposed methodologies are very close to the previous case study, confirming the effectiveness of the approach.

**Figure 12:**
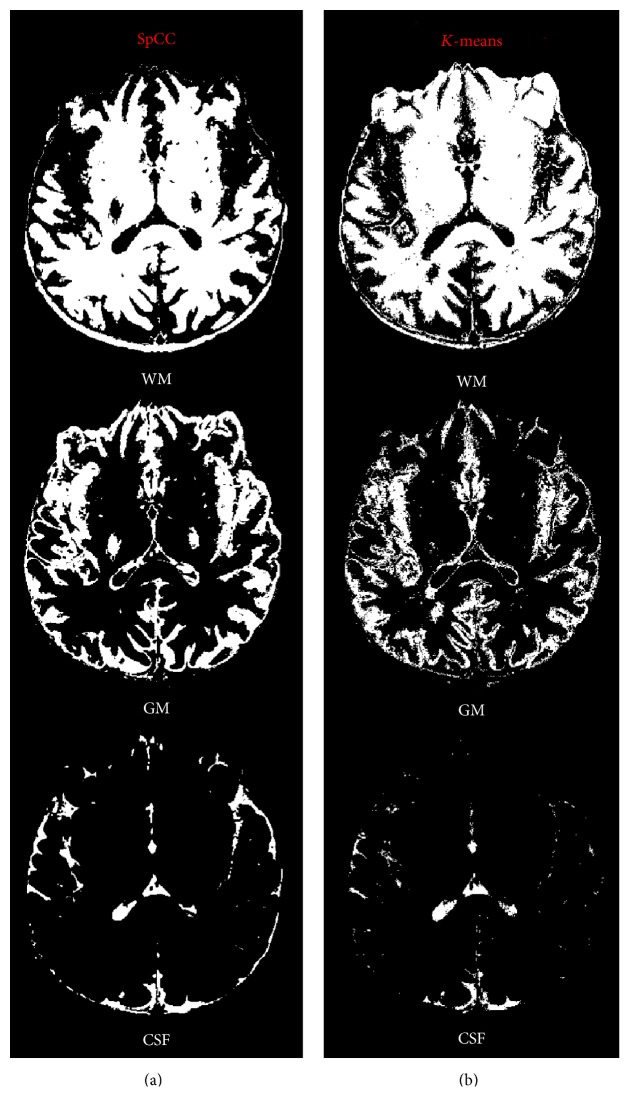
Classification results of the proposed approach based on SpCC (a) and of *K*-means technique (b).

Moving to real dataset, among the three presented techniques, only SpCC methodology has been considered, being the most accurate, and compared with *K*-means approach. Results reported in Figure 12 show that both algorithms are able to detect the three regions: WM, GM, and CSF. *K*-means globally produces low regularized segmentation regions, with some classification errors in the retroocular region (GM map) and in the temporal area (CSF map). Moving to SpCC, segmented regions appear much more realistic, more regular, and without isolated spots. SpCC shows effective results especially in the retroocular region in the GM map and in the proximity of the insula region concerning CSF. However two WM nuclei have erroneously been included in the GM region. That said, it has to be underlined that the proposed unsupervised approach has substantial room for improvement if combined with more sophisticated regularization criteria, but it is evident how the proposed multidimensional distance metric is promising.

## 4. Conclusions

Within this paper a novel unsupervised approach for joint segmentation and classification in brain MRI has been presented. The peculiarity of the approach consists in detection criteria applied to the estimated proton density (*ρ*) and relaxation times (*T*_1_ and *T*_2_) maps, instead of to the acquired gray level image. It has to be pointed out that the effectiveness of the method is strictly related to the accuracy of adopted relaxation times estimator, which depends on several issues such as the number of images, the noise intensity, and the acquisition scheme [10, 32]. Moreover, the need of multiple images and of an estimation step implies longer acquisition and computational times. After estimating the physical parameters from multiple scans, the segmentation is performed in a statistical framework. The aim of the paper is to show the feasibility of segmenting brain based on proton density and relaxation times. In particular, the paper focuses on the effectiveness of considering a 3D statistical metric for evaluating distances instead of a 1D one.

Results, validated on simulated datasets, are interesting and promising, greatly improving the detection rate with respect to a classical minimum distance based technique and other widely adopted segmentation methodologies. Moreover, results obtained on the real brain datasets appear reliable and consistent. A further refinement on the regularization criteria can be investigated in the future.

## Figures and Tables

**Table 1:** Real dataset, imaging protocol details.

MRI scanner	Philips Achieva
Field intensity	3.0 T
Coil	Bird cage, 8 channels
Sequence	Spin Echo
FOV	230 × 230 mm
Voxel size	0.45 × 0.45 × 3 mm
Image resolution	512 × 512 pixels
Number of images	4
Echo times [ms]	82.45, 82.45, 200, 200
Repetition times [ms]	700, 3500, 700, 3500

## References

[1] Kamber M., Shinghal R., Collins D. L., Francis G. S., Evans A. C. (1995). Model-based 3-D segmentation of multiple sclerosis lesions in magnetic resonance brain images. *IEEE Transactions on Medical Imaging*.

[2] Kaus M. R., Warfield S. K., Nabavi A., Black P. M., Jolesz F. A., Kikinis R. (2001). Automated segmentation of MR images of brain tumors. *Radiology*.

[3] Tanabe J. L., Amend D., Schuff N. (1997). Tissue segmentation of the brain in Alzheimer disease. *American Journal of Neuroradiology*.

[4] Widz S., Revett K., Ślezak D. (2004). An automated multi-spectral MRI segmentation algorithm using approximate reducts. *Rough Sets and Current Trends in Computing*.

[5] Clarke L. P., Velthuizen R. P., Camacho M. A. (1995). MRI segmentation: methods and applications. *Magnetic Resonance Imaging*.

[6] Clarke L. P., Velthuizen R. P., Phuphanich S., Schellenberg J. D., Arrington J. A., Silbiger M. (1993). MRI: stability of three supervised segmentation techniques. *Magnetic Resonance Imaging*.

[7] Li C., Huang R., Ding Z., Gatenby J. C., Metaxas D. N., Gore J. C. (2011). A level set method for image segmentation in the presence of intensity inhomogeneities with application to MRI. *IEEE Transactions on Image Processing*.

[8] Torres W., Martín-Landrove M., Paluszny M., Figueroa G., Padilla G. Tumor segmentation of multiecho MR T2-weighted images with morphological operators.

[9] Ma D., Gulani V., Seiberlich N. (2012). Magnetic resonance fingerprinting. *Nature*.

[10] Cho Z.-H., Jones J., Singh M. (1993). *Foundations of Medical Imaging*.

[11] Wright G. A. (1997). Magnetic resonance imaging. *IEEE Signal Processing Magazine*.

[12] Wang Y., Lei T. Statistical analysis of MR imaging and its applications in image modeling.

[13] Baselice F., Ferraioli G., Shabou A. (2010). Field map reconstruction in magnetic resonance imaging using Bayesian estimation. *Sensors*.

[14] Eggers H., Knopp T., Potts D. (2007). Field inhomogeneity correction based on gridding reconstruction for magnetic resonance imaging. *IEEE Transactions on Medical Imaging*.

[15] Baselice F., Ferraioli G., Pascazio V. (2010). Relaxation time estimation from complex magnetic resonance images. *Sensors*.

[16] Baselice F., Ferraioli G., Pascazio V. (2015). A Bayesian approach for relaxation times estimation in MRI. *Magnetic Resonance Imaging*.

[17] Baselice F., Ferraioli G., Grassia A., Pascazio V. (2014). Optimal configuration for relaxation times estimation in complex spin echo imaging. *Sensors*.

[18] Wansapura J. P., Holland S. K., Dunn R. S., Ball W. S. (1999). NMR relaxation times in the human brain at 3.0 Tesla. *Journal of Magnetic Resonance Imaging*.

[19] Zhan T., Zhang J., Xiao L., Chen Y., Wei Z. (2013). An improved variational level set method for MR image segmentation and bias field correction. *Magnetic Resonance Imaging*.

[20] Chen Y., Zhang J., Yang J. (2012). An anisotropic images segmentation and bias correction method. *Magnetic Resonance Imaging*.

[21] Li S. Z. (2001). *Markov Random Field Modeling in Image Analysis*.

[22] Guerquin-Kern M., Lejeune L., Pruessmann K. P., Unser M. (2012). Realistic analytical phantoms for parallel magnetic resonance imaging. *IEEE Transactions on Medical Imaging*.

[23] Baselice F., Caivano R., Cammarota A., Ferraioli G., Pascazio V. (2014). *T*
_1_ and *T*
_2_ estimation in complex domain: first results on clinical data. *Concepts in Magnetic Resonance Part A*.

[24] Baselice F., Caivano R., Cammarota A., Ferraioli G., Pascazio V. Relaxation times estimation in MRI.

[25] Adams R., Bischof L. (1994). Seeded region growing. *IEEE Transactions on Pattern Analysis and Machine Intelligence*.

[26] Kapur J. N., Sahoo P. K., Wong A. K. C. (1985). A new method for gray-level picture thresholding using the entropy of the histogram. *Computer Vision, Graphics, and Image Processing*.

[27] MacQueen J. (1967). Some methods for classification and analysis of multivariate observations. *Proceedings of the Fifth Berkeley Symposium on Mathematical Statistics and Probability*.

[28] Jaccard P. (1901). Étude comparative de la distribution orale dans une portion des alpes et des jura. *Bulletin de la Société Vaudoise des Sciences Naturelles*.

[29] Dice L. R. (1945). Measures of the amount of ecologic association between species. *Ecology*.

[30] Sørensen T. (1948). A method of establishing groups of equal amplitude in plant sociology based on similarity of species and its application to analyses of the vegetation on Danish commons. *Biologiske Skrifter*.

[31] Kwan R. K.-S., Evans A. C., Pike B. (1999). MRI simulation-based evaluation of image-processing and classification methods. *IEEE Transactions on Medical Imaging*.

[32] D'Arco M., Genovese M., Napoli E., Vadursi M. (2014). Design and implementation of a preprocessing circuit for bandpass signals acquisition. *IEEE Transactions on Instrumentation and Measurement*.

